# 

**Published:** 1880-06

**Authors:** 


*CONTENTS.*

| | Page |
|---|---|
| Sea Sickness | 149 |
| Checking Perspiration | 151 |
| Remedy for a Cough | 153 |
| Throat Ail | 156 |
| Influences | 158 |
| Agriculture | 159 |
| Summering in the Country | 159 |
| Punishing Children | 163 |
| Checking Perspiration | 164 |
| Popular Fallacies | 166 |
| Exercise | 169 |
| Health for Children | 171 |
| Premium on Babies | 172 |
| Face Painting and Enameling | 175 |
| Wrecked Clergymen | 176 |
| Marrying Well | 177 |

